# Predicting radioiodine therapy response in advanced and recurrent thyroid cancer

**DOI:** 10.1530/ERC-26-0253

**Published:** 2026-09-29

**Authors:** Vincenzo Condello, Myrsini Strati, Daniel Thor, Catharina Larsson, C Christofer Juhlin, Joachim N Nilsson

**Affiliations:** ^1^ Department of Oncology-Pathology, Karolinska Institutet, Stockholm, Sweden; ^2^ Department of Nuclear Medicine and Medical Physics, Karolinska University Hospital, Stockholm, Sweden; ^3^ Department of Clinical Pathology and Cancer Diagnostics, Karolinska University Hospital, Stockholm, Sweden; ^4^ Department of Molecular Medicine and Surgery, Karolinska Institutet, Stockholm, Sweden

**Keywords:** thyroid cancer, radioiodine, immunohistochemistry, genetics

## Abstract

Radioiodine therapy of differentiated thyroid cancer is effective and possibly curative in patients with metastatic lesions that retain iodine avidity. However, in clinical practice, iodine avidity in any metastatic site is usually unknown at the point of initial treatment. Avidity prediction could enable more tailored initial radioiodine treatment. This work aimed to establish the best predictive factors for iodine avidity and subsequent treatment response. Thirty-one patients with metastatic or recurrent thyroid cancer sites in which iodine avidity could be assessed by image-based analysis were included. In tissue specimens from primary surgery, immunohistochemical expression of thyroglobulin (Tg), thyroid peroxidase (TPO), sodium-iodide symporter (NIS), and Ki67 and mutational status of *BRAF*, *RAS*, and the *TERT* promoter were analyzed. Biochemical and structural responses were assessed by serum Tg response and radiological evaluation. High-risk histology (widely invasive follicular, tall cell subtype papillary, differentiated high-grade, or poorly differentiated thyroid carcinoma) combined with the expression of TPO, Tg, and cytoplasmic NIS performed well in predicting treatment response and iodine avidity. Approximately half of the variation between patients was explained by those variables, outperforming pT stage and mutational status. TPO and Tg expression were the largest contributors to prediction modeling. Radioiodine treatment response and iodine avidity in thyroid cancer can be predicted from histopathological analysis of the primary tumor. High-risk histology and the expression of TPO, Tg, and NIS were robust and informative predictors. This may be used to adapt treatment strategy in adjuvant and metastatic therapy settings.

## Introduction

Radioiodine (RAI) has been used for eight decades in the treatment of differentiated thyroid cancer. For most of that time, treatment has been administered without prior knowledge of its likelihood of effectiveness in individual patients. The underlying potency of the treatment, together with a favorable low-toxicity profile, even when given without adjusted dosage, has produced impressive results and made it a cornerstone in thyroid cancer treatment (1, 2).

Two conditions are required for RAI therapy to be effective. The first requirement is that there has to be tumor tissue left to treat, given that most patients are cured by surgery alone (3). This fact, combined with the excellent prognosis for the great majority of patients with papillary (PTC) and follicular thyroid carcinoma (FTC), is the main reason that indications for RAI treatment are narrowing (4, 5). Consequently, RAI is increasingly reserved for patients with macroscopic residual tumor or patients at high risk of recurrence in both American and European guidelines (6, 7). While the TNM staging system stratifies patients according to disease-specific mortality, the potential benefit of RAI therapy is more closely aligned with recurrence risk stratification systems, such as the ATA guidelines (8). The second requirement for effective treatment is iodine avidity; tumors need to retain much, or at least some, of the intricate iodine metabolism of healthy thyroid cells (9). Iodine avidity and, consequently, tumor radiation dose from RAI vary widely. This means that administration of RAI can result in tumor doses far below the levels required for successful treatment, as illustrated in a 2025 review of dose–effect relationships in thyroid cancer (10). With upfront knowledge of iodine avidity, both patient selection and dosage in the treatment of advanced and high-risk thyroid cancer could be improved.

Previous research has shown that in differentiated thyroid cancer, *BRAF^V600E^*-mutant tumors are associated with reduced iodine avidity – further decreased when accompanied by concomitant *TERT* promoter mutations – whereas *RAS*-mutant-driven tumors are more likely to retain avidity (11, 12, 13, 14). This effect is mediated, in part, by constitutive MAPK pathway activation, which suppresses sodium-iodide symporter (NIS) expression, the key protein responsible for iodine uptake (15). Conversely, targeted inhibition of BRAF or MEK can restore NIS expression and enhance iodine uptake (16, 17). However, basolateral plasma membrane localization of NIS, required for effective iodide transport and indicative of avidity, appears uncommon and difficult to detect with immunohistochemistry in thyroid cancer (18, 19, 20, 21).

Therefore, links between iodine avidity and expression of other iodine-related proteins such as TPO and Tg are of interest but have been less extensively studied than NIS. Out of three studies that compared iodine uptake with immunohistochemical Tg expression, only one showed a convincing relationship (22, 23, 24). TPO has not explicitly been studied in relation to iodine avidity, but its expression seems to be positively correlated with differentiation in tumors (25, 26). However, recently both Tg and TPO expression have been linked to iodine avidity in a previous prospective cohort including data on *ex vivo* radioiodine uptake (27, 28). It is known that oncocytic thyroid carcinoma and poorly differentiated thyroid carcinoma (PDTC) have lower avidity than FTC and PTC and are often RAI-refractory from the onset (29, 30, 31). With respect to separating patients with high and low iodine avidity, the value of TNM appears to be much lower (28).

While molecular alterations and differentiation status provide important contextual information, they lack sufficient sensitivity and specificity with regard to avidity. Immunohistochemical assessment of iodine-handling proteins has so far proven technically challenging and biologically inconsistent. As a result, iodine avidity continues to be determined largely empirically in clinical practice. More extensive pathology-based prediction could improve patient stratification prior to RAI administration.

In this work, we comprehensively analyzed a retrospective cohort to determine which histologic, immunohistochemical, and molecular features best predict iodine avidity and treatment response.

## Materials and methods

An overview of the study design is presented in Fig. 1.

**Figure 1:**
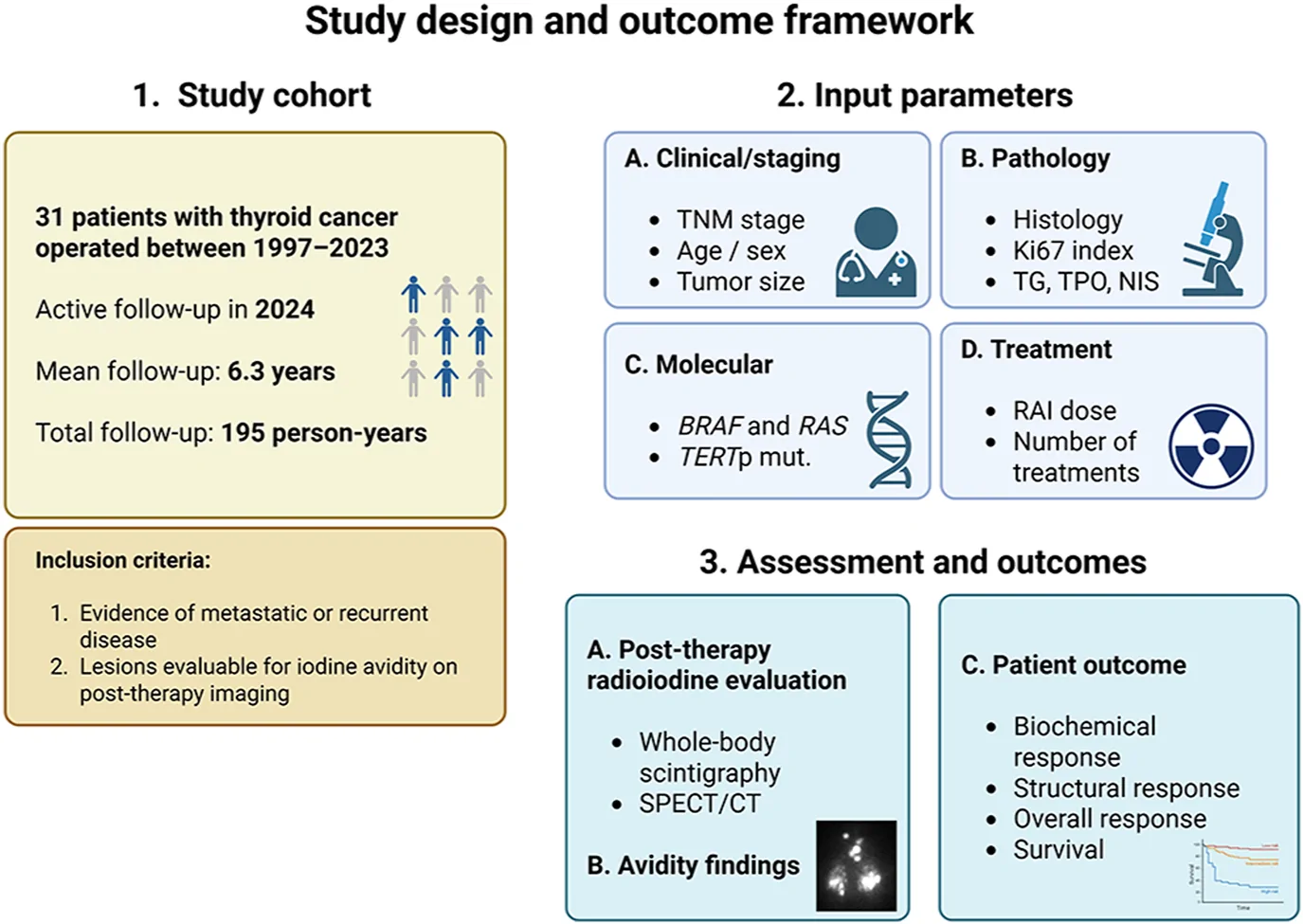
Overview of the study design. A full-color version of this figure is available at https://doi.org/10.1530/ERC-26-0253.

### Study design and inclusion

The study is a retrospective analysis of data from surgery, pathology, and imaging in 31 patients treated with thyroidectomy, hormone suppression, and RAI therapy. All patients had undergone thyroidectomy at Karolinska University Hospital, Stockholm, Sweden, in either one or two steps, between 1997 and 2023 and had active follow-up in 2024. Crucially, only patients with confirmed subsequent metastatic disease that could be evaluated on RAI scans were included in the study. All participants provided written informed consent, and the study had ethical approval.

### RAI treatment

Following thyroidectomy, patients received RAI within three months, with administered activities in accordance with national guidelines, generally 3.7–7.4 GBq per administration. Treatment was performed under exogenous TSH stimulation and was repeated at the discretion of the treating physician and the multidisciplinary tumor board when considered beneficial in the individual case. All patients were maintained on thyroid hormone suppression therapy.

### Image analysis and dosimetry

Functional imaging, including whole-body scintigraphy and SPECT/CT scans, was acquired seven days after administration of RAI. In a subset of six patients, serial SPECT/CT imaging at two or three time points after RAI administration allowed for dosimetric evaluation. In these patients, tumor lesion activity was estimated from attenuation-corrected SPECT images, and delineation of the corresponding lesion volume was performed on CT images. The resulting activity concentrations were multiplied by an in-house MCNP-simulated volume-dependent conversion factor to obtain dose rates. By monoexponential fitting and integration of the dose–rate curve, the absorbed dose was calculated. Whole-body scintigraphy scans, together with SPECT/CT images when available, after the first RAI treatment were assessed for iodine avidity. In the images, mean pixel counts in a region of interest in the tumor were divided by the mean pixel counts in a region of interest in either the brain or back muscle, whichever was within the scan field of view and clear of any known metastatic lesions. Although no standardized approach is established, the method used in the present study was consistent with previously described methods (32). This tumor-to-normal ratio was subsequently used as a measure of iodine avidity in tumors.

### Response evaluation

Biochemical response to RAI treatment was assessed by evaluating changes in serum Tg levels between administration of RAI and the follow-up measurement obtained closest to six months after treatment. Serum Tg was measured under TSH suppression therapy. In the analysis, change in Tg was assessed after the second RAI administration because using the first RAI would risk confounding by the size and activity of any thyroid remnant. In three cases (3/31), Tg levels were low or undetectable, but anti-Tg antibodies were present. For those patients, changes in antibody levels were used instead. Patients were categorized as biochemical responders only if their serum Tg levels decreased or remained stable (maximum 10% increase) after every RAI treatment.

Structural response was evaluated based on anatomical and functional imaging and clinical examination. Response to the first RAI administration was initially categorized as ‘complete response’, ‘partial response’, ‘stable disease’, or ‘progressive disease’ based on radiology reports from radioiodine scans and any other cross-sectional imaging.

Patients were categorized as ‘RAI responders’, an aggregate outcome variable, if they met either of the following criteria: (i) structural and functional ‘complete response’ and no detectable serum Tg after initial RAI treatment and therefore received only one administration of RAI; or (ii) structural and functional ‘partial response’ that never became ‘progressive disease’ during follow-up, combined with undetectable, decreasing, or stable (maximum 10% increase) serum Tg levels after each RAI treatment.

A sensitivity analysis was performed by analyzing (modeling) the dataset with the following changes: (i) excluding patients with only lymph node metastases; (ii) changing the definition of structural response for patients with ‘stable disease’ from RAI nonresponders to responders; and (iii) excluding any patients with PDTC and differentiated high-grade thyroid carcinoma (DHGTC).

### Histopathology

Original pathology reports were assessed and used for analysis in the study. The classifications were according to the most recent WHO classification at the time. In one case, a tumor progressed from widely invasive FTC (wiFTC) to PDTC – the initial wiFTC diagnosis was used in the present study. In two separate cases, the initial diagnosis was a benign entity despite subsequent development of distant metastases. In both those cases, tissue specimens were reexamined and reclassified according to the 2022 WHO classification as follicular thyroid tumors of uncertain malignant potential (FT-UMP) (33). High-risk histology was defined as either PDTC, DHGTC, wiFTC, or tall cell subtype PTC (tcPTC). No widely invasive oncocytic, hobnail PTC, or other high-risk histology subtypes were present in the cohort.

### Immunohistochemistry

IHC staining for Tg, Ki67, NIS, and TPO was performed on 4-μm-thick slices of formalin-fixed, paraffin-embedded (FFPE) tissue using previously described methodology (27, 28). Specifically, staining for Tg and Ki67 was carried out on the Ventana BenchMark Ultra platform using a routine clinical protocol. Briefly, FFPE tissues were deparaffinized in xylene and rehydrated through graded ethanol. Antigen retrieval was performed for 64 min using Ultra CC1, followed by incubation with the primary antibodies at room temperature for 32 min. After incubation with the secondary antibody, signal detection was carried out using the UltraView DAB detection system as part of the clinical routine (34).

Staining for NIS and TPO was performed manually as previously reported (28). Briefly, tissue sections were deparaffinized and rehydrated, followed by antigen retrieval in citrate buffer (pH 6.0) for 15 min at 110°C using a Decloaking Chamber (Biocare Medical, California, USA). Endogenous peroxidase activity was quenched by incubation with 0.15% hydrogen peroxide for 30 min at room temperature, followed by a 30-min blocking step with 1% bovine serum albumin (BSA). Tissue sections were incubated with the primary antibodies overnight at 4°C in a humidified chamber. A biotinylated secondary antibody was then applied for 30 min at room temperature, followed by incubation with an avidin–biotin peroxidase complex (Vectastain ELITE ABC HRP kit, PK-6100; Vector Laboratories, California, USA) for an additional 30 min. Immunoreactivity was visualized using a diaminobenzidine (DAB) substrate (Vector DAB SK-4100; Vector Laboratories, California, USA) for 3 min. Sections were counterstained with Mayer’s hematoxylin for 1 min, dehydrated through graded alcohols, cleared in xylene, and coverslipped using Pertex (Histolab, Sweden).

Staining was evaluated by an experienced endocrine pathologist and scored according to the percentage of cells expressing each marker. Specifically, NIS and TPO were scored in bins of 0–10, 10–50, 50–75, or 75–100%. In the analysis, these bins were transformed to values of either 0, 30, 60, or 90% to allow the use of expression as a continuous variable in the prediction modeling. Tg expression was based on clinical routine IHC and was scored in steps of 5%. Ki67 was scored as a continuous percentage.

### Mutation analyses

Genomic DNA was extracted from frozen and FFPE tissues using the DNeasy Blood and Tissue kit (Qiagen, Germany) according to the manufacturer’s instructions. Hotspot mutations were screened for in *BRAF* (exon 15, codon 600), *RAS* genes (*NRAS, HRAS, KRAS, *exons 2–3, codons 12–13 and 61), and the *TERT* promoter (C228T and C250T) by direct sequencing according to standard procedures (35, 36).

### Statistical analysis

All statistical analyses were performed using R (version 4.2.2, r-project.org). Statistical tests for correlation used the Pearson correlation coefficient (cor.test function in R), and differences in proportions were tested using Fisher’s exact test (fisher.test function in R). Differences in continuous values between dichotomous groups were assessed using Welch’s *t*-test (*t*.test function in R).

Models for iodine avidity and biochemical response were constructed through linear multivariate regression (lm function in R). Modeling diagnostics were conducted as follows: normality of residuals was assessed using quantile–quantile plots; collinearity was assessed using variance inflation factors (VIF function in R); and scedasticity was assessed using the Goldfeld–Quand test (gqtest function in R). Models were evaluated with adjusted *R*^2^. Prediction models for structural response and RAI response were constructed with binomial logistic regression (glm function in R). The logistic regression was evaluated using McFadden’s pseudo-*R*^2^ (calculated manually) and the Hosmer–Lemeshow test (hoslem.test function in R). Multivariate modeling aimed to find a prediction model with robust prediction for several endpoints (tumor-to-normal ratio, biochemical response, and RAI response). The model was selected according to performance (based on evaluation of adjusted and pseudo-*R*^2^), robustness (the model had to be well-performing for all endpoints), and simplicity (few input variables).

Two values of primary tumor size and one value each of TPO and NIS expression were imputed before performing regression modeling. Data imputation was performed using a random forest method (missForest function in R). In analyses involving tumor-to-normal ratios and serum Tg changes, values were log-transformed to approach normality.

## Results

A total of 31 patients were included in the analysis, spanning 195 person-years of follow-up; patients were followed for a mean of 6.3 years. Patient characteristics and treatment details are summarized in Table 1.

**Table 1:** Clinicopathological characteristics and treatment details of the 31 patients.

	All patients (*n* = 31)	RAI response	
		Yes (*n* = 8)	No (*n* = 23)
**Age**			
Median (range), yrs	64 (28–84)	56 (28–71)	67 (29–84)
**Sex**			
Female/male	19/12	3/5	16/7
**Tumor type**			
PTC	13	5	8
FTC	9	1	8
DHGTC	3	0	3
PDTC	4	0	4
FT-UMP	2	2	0
**pT stage**			
1a	2	1	1
1b	5	0	5
2	5	4	1
3a	11	3	8
3b	6	0	6
x	2	0	2
**N stage**			
0	1	0	1
1a	1	1	0
1b	13	4	9
x	16	3	13
**M stage**			
0	11	4	7
1	20	4	16
**Mutational status**			
*RAS/RAS + TERT*p	3/8	0/3	3/5
*BRAF^V600E^/BRAF^V600E^* + *TERT*p	9/2	1/0	8/2
*TERT*p	1	0	1
Neither	8	4	4
**Metastatic site**			
Distant (lungs, bones)	23	5	18
Lymph nodes	7	3	4
Mediastinal	1	0	1
**Number of RAI**			
Median (range)	2 (1–6)	2 (1–5)	2 (1–6)
**Total RAI activity**			
Median (range) (GBq)	11 (1–40)	9 (1–35)	13 (4–40)
**Follow-up outcome**			
Progression	16 (52%)	0 (0%)	16 (70%)
Recurrence	7 (23%)	0 (0%)	7 (30%)
Disease-related death	6 (19%)	0 (0%)	6 (30%)

PTC, papillary thyroid carcinoma; FTC, follicular thyroid carcinoma; DHGTC, differentiated high-grade thyroid carcinoma; PDTC, poorly differentiated thyroid carcinoma; FT-UMP, follicular thyroid tumor of uncertain malignant potential; RAI, radioiodine; yrs, years; GBq, gigabecquerel.

### Iodine avidity

Tumor-to-normal ratios varied widely, ranging from 1 to 3,000 (median: 20; geometric mean: 207). In 42% of patients (13/31), no distinct iodine uptake was observed (defined in this work as ratios below 4, five-fold lower than the median value), indicating low or no iodine avidity. The tumor-to-normal ratio data are included in Figs 2, 3, and 4.

**Figure 2:**
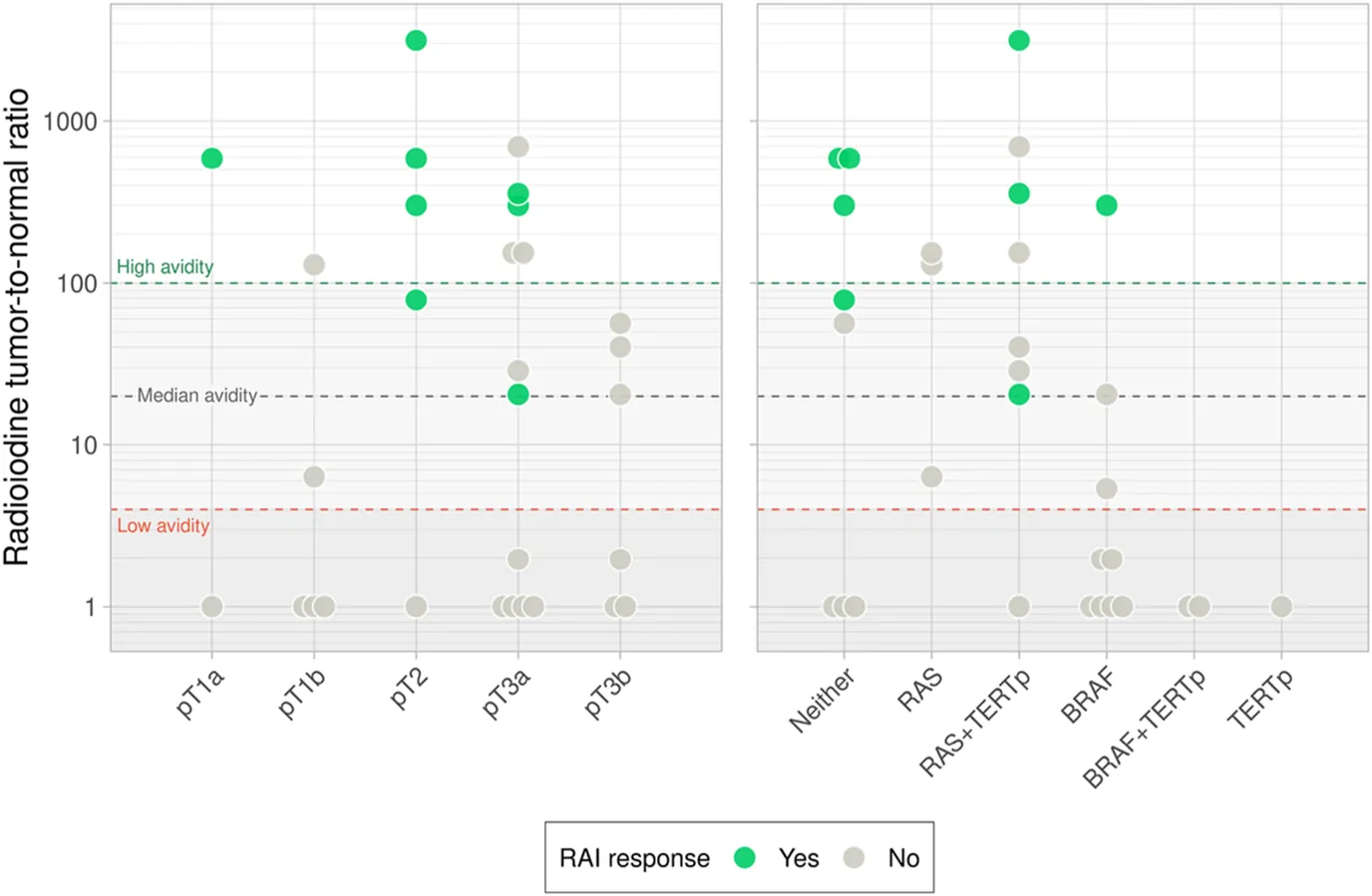
Treatment response and tumor-to-normal ratios on a logarithmic scale according to (left) pT stage and (right) mutational status (*n* = 31). No clear differences in rates of response or tumor-to-normal ratios were found according to pT stage or mutational status. The median tumor-to-normal ratio (at 20) is highlighted, along with ‘high’ (at 100) and ‘low’ (at 4) ratios, set five times higher and lower than the median value. A full-color version of this figure is available at https://doi.org/10.1530/ERC-26-0253.

**Figure 3:**
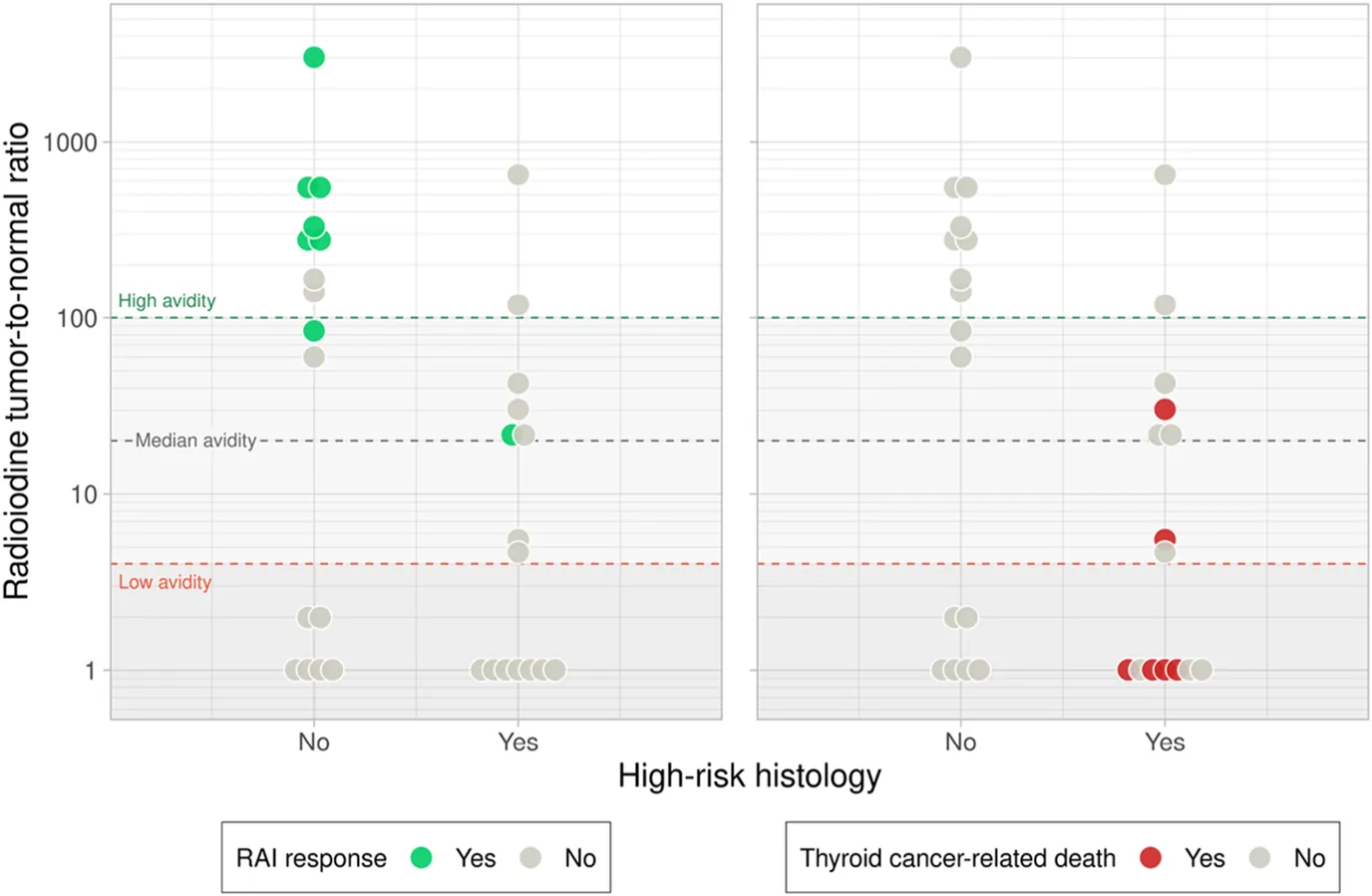
Tumor-to-normal ratio on a logarithmic scale according to high-risk histology categorization. Data are shown colored for both (left) RAI response and (right) disease-related death according to high-risk histology (*n* = 31). There was a significantly lower chance of treatment response and higher risk of death in the high-risk histology group. The median tumor-to-normal ratio (at 20) is highlighted, along with ‘high’ (at 100) and ‘low’ (at 4) ratios, set five times higher and lower than the median value. A full-color version of this figure is available at https://doi.org/10.1530/ERC-26-0253.

**Figure 4:**
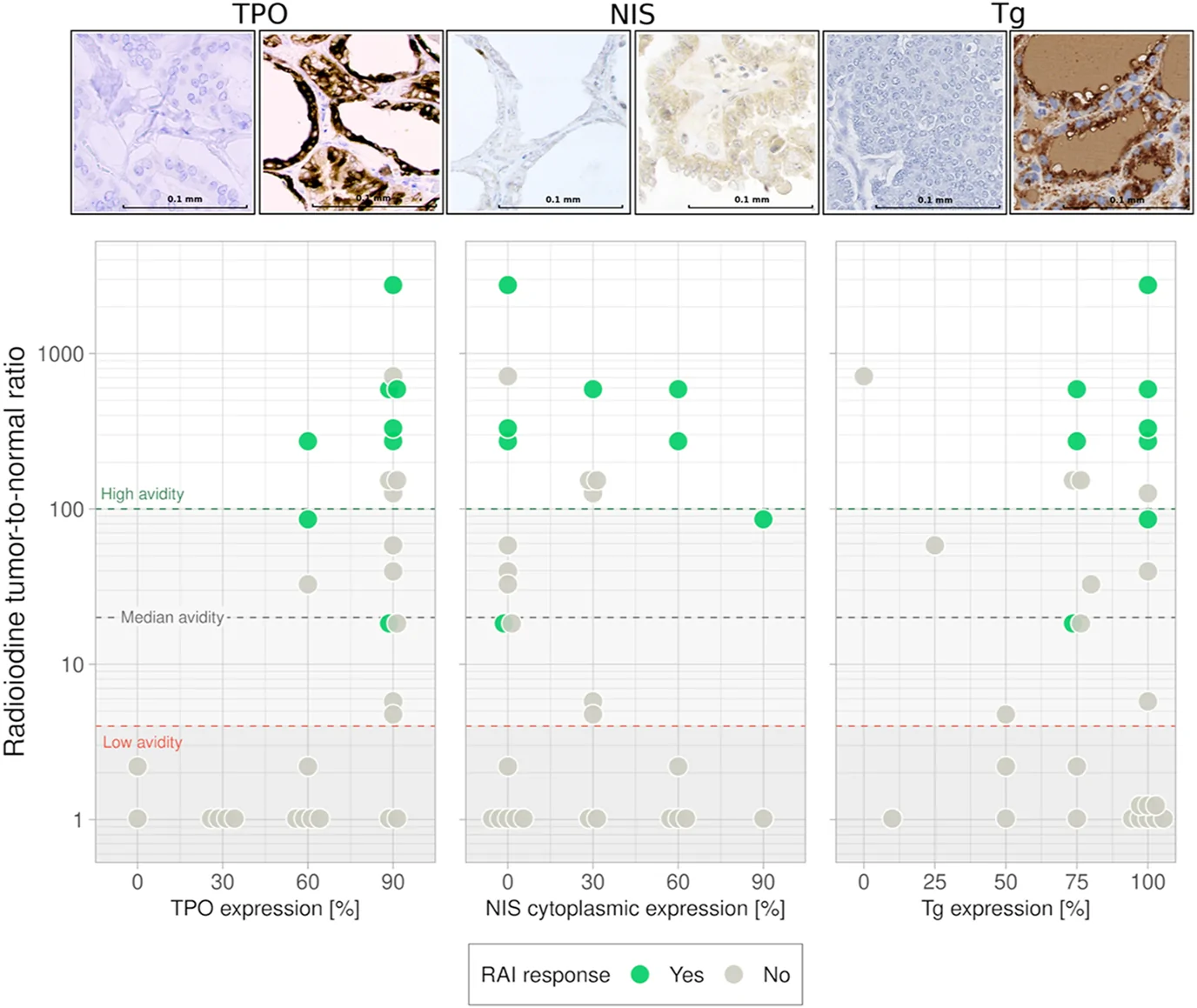
Treatment response and tumor-to-normal ratio on a logarithmic scale according to (left) TPO, (middle) cytoplasmic NIS, and (right) Tg immunohistochemical expression (*n* = 31). Above the graphs are representative immunohistochemistry scans with low and high expression of the respective proteins. There was a significant correlation between TPO expression and tumor-to-normal ratio. No significant association between treatment response and any of the markers was observed. The median tumor-to-normal ratio (at 20) is highlighted, along with ‘high’ (at 100) and ‘low’ (at 4) ratios, set five times higher and lower than the median value. A full-color version of this figure is available at https://doi.org/10.1530/ERC-26-0253.

Comparison of scintigraphy signal tumor-to-normal ratios with detailed dosimetric analysis using SPECT/CT in the six patients for whom it was available showed excellent agreement (see Fig. S1 of the Supplementary materials). This supported the relevance of tumor-to-normal ratios as a proxy for absorbed dose, and tumor-to-normal ratios were used in all subsequent analyses.

### Biochemical response

The primary biochemical outcome variable, change in serum Tg after the second RAI administration, was available in 65% of patients (20/31). The remaining patients had the following reasons for not receiving a second RAI: no uptake on the RAI post-treatment scan (five patients); RAI response after the first RAI treatment (three patients); or progressive disease despite RAI uptake (three patients).

Biochemical response ranged from a 9-fold decrease to a 15-fold increase in serum Tg at six months after RAI. The median change was a 1.4-fold decrease. Tg change correlated well with tumor-to-normal ratios, with a correlation coefficient of −0.52 (95% CI: −0.78 to −0.09; Supplementary Fig. S2).

### Structural response

Initial structural response was possible to evaluate in 84% of patients (26/31). Among evaluable patients, two were initially categorized as ‘complete response’, six as ‘partial response’, eight as ‘stable disease’, and ten as ‘progressive disease’. The remaining five patients had lesions extirpated surgically within months after RAI, precluding any analysis of structural change after RAI treatment. While 62% of patients (16/26) did not have ‘progressive disease’ after initial RAI, only 39% of patients subsequently remained outside this category.

### RAI response

Out of all 31 patients, 26% (8/31) had both sustained biochemical and structural responses and were categorized as ‘RAI responders’. The association between RAI response, pT stage, and genetic markers is shown in Fig. 2. pT stage did not clearly predict RAI response, and no significant differences were observed according to mutational status.

A significantly lower proportion of patients with high-risk histology were RAI responders compared with those without high-risk histology (7 vs 44%; 95% CI: 0–32% in the high-risk histology group). The relationship between tumor-to-normal ratio, RAI response, and death versus high-risk histology is shown in Fig. 3. During follow-up, 19% of patients (6/31) died from their metastatic thyroid cancer (Fig. 3). All deaths occurred in patients with high-risk histology, corresponding to 43% disease-specific mortality within that group (6/14), whereas no deaths were observed among patients without high-risk histology (0/17), representing a significant difference (odds ratio 95% CI: 1.87 to Inf for the high-risk group).

Across histological types included in the cohort (PTC, FTC, FT-UMP, DHGTC, and PDTC), iodine avidity varied widely. Between FTC and PTC, no significant differences in iodine avidity or RAI response rate were observed (Supplementary Fig. S3). Due to the small number of cases of FT-UMP, DHGTC, and PDTC, no analysis was performed for these entities. The fact that both tumors classified as FT-UMP recurred with distant metastases contradicts the classification’s uncertain malignant potential. Instead, they could be considered clinically malignant follicular tumors, which is not an established classification. The authors are aware of this discrepancy while reporting both cases as FT-UMP throughout, as this was the correct histological designation for both cases because no invasive foci were seen upon histological examination.

There was a strong correlation between tumor-to-normal ratio and TPO expression (correlation coefficient: 0.60; 95% CI: 0.31–0.79). In contrast, no significant correlation was observed for cytoplasmic NIS, membranous NIS, Ki67, or Tg. Furthermore, none of TPO, Ki67, Tg, or NIS expression alone clearly correlated with RAI response. Data for TPO, Tg, and cytoplasmic NIS are shown in Fig. 4 (data for Ki67 are shown in Supplementary Fig. S4).

### Multivariate modeling

Multivariate linear and logistic models for predicting tumor-to-normal ratio, biochemical response, and RAI response were constructed and compared. Based on benchmarking with adjusted *R*^2^ or pseudo-*R*^2^, the best-performing and most robust model used expression of TPO, Tg, and cytoplasmic NIS, and high-risk histology. The resulting model was designed based on mechanistic reasoning related to the predictors and an aim to limit the number of variables as much as possible. This model showed consistently strong performance across tumor-to-normal ratio (adjusted *R*^2^ = 0.47), biochemical response (adjusted *R*^2^ = 0.46), and RAI response (pseudo-*R*^2^ = 0.35). The results persisted when patients with lymph node metastases (7/31 patients) were excluded from the analysis across tumor-to-normal ratio (adjusted *R*^2^ = 0.70), biochemical response (adjusted *R*^2^ = 0.46), and RAI response (pseudo-*R*^2^ = 0.28). When excluding patients with PDTC and DHGTC from the analysis (also 7/31 patients), the modeling results similarly persisted for tumor-to-normal ratio (adjusted *R*^2^ = 0.49), biochemical response (adjusted *R*^2^ = 0.46), and RAI response (pseudo-*R*^2^ = 0.28). When changing the definition of ‘RAI responder’ to include ‘stable disease’ as structural response, the model performed slightly worse (RAI response pseudo-*R*^2^ = 0.19). The logistic regression results for including ‘stable disease’ in the responder group are shown in Supplementary Table S1. In summary, this corresponds to the model explaining approximately 50% of variation in avidity and treatment outcome in the cohort. The models had favorable residual distribution and variance and showed no signs of multicollinearity. More details on modeling results for avidity and treatment response for the full cohort are shown in Table 2.

**Table 2:** Multivariate modeling results for the best-performing and most robust model.

Predicted endpoint	Predictive variable	exp(β)	95% CI	
**Iodine avidity (linear regression)**		(Multiplicative factor)		Adjusted *R*^2^: 0.47
Tumor-to-normal ratio	Intercept: 0.84			
	High-risk histology (yes)	0.12	0.02–0.52**	
	TPO expression (per 10%)	1.78	1.37–2.31***	
	Cytoplasmic NIS expression (per 10%)	0.76	0.57–1.00	
	Tg expression (per 10%)	1.09	0.83–1.44	
**Biochemical response (linear regression)**		(Multiplicative factor)		Adjusted *R*^2^: 0.46
Serum Tg change (%)	Intercept: two-fold increase			
	High-risk histology (yes)	1.67	0.64–4.33	
	TPO expression (per 10%)	0.87	0.71–1.07	
	Cytoplasmic NIS expression (per 10%)	1.46	1.18–1.82**	
	Tg expression (per 10%)	0.87	0.71–1.06	
**RAI response (logistic regression)**		(Odds ratio)		Pseudo-*R*^2^: 0.35
Sustained biochemical and structural response	Intercept: odds 0.0024			
	High-risk histology (yes)	0.07	0.00–0.64*	
	TPO expression (per 10%)	1.50	1.02–2.27*	
	Cytoplasmic NIS expression (per 10%)	0.92	0.60–1.36	
	Tg expression (per 10%)	1.46	0.92–2.97	

The numbers in the exp(β) columns are the estimated multiplicative risk and odds modifiers for the respective endpoints.

*P*-values: *<0.05; **<0.01; ***<0.001.

In the logistic regression model for RAI response, TPO and Tg expression were the most influential predictors, together accounting for more than 70% of the predictive power of the model. The marginal contributions for the four predictive variables are illustrated in Fig. 5.

**Figure 5:**
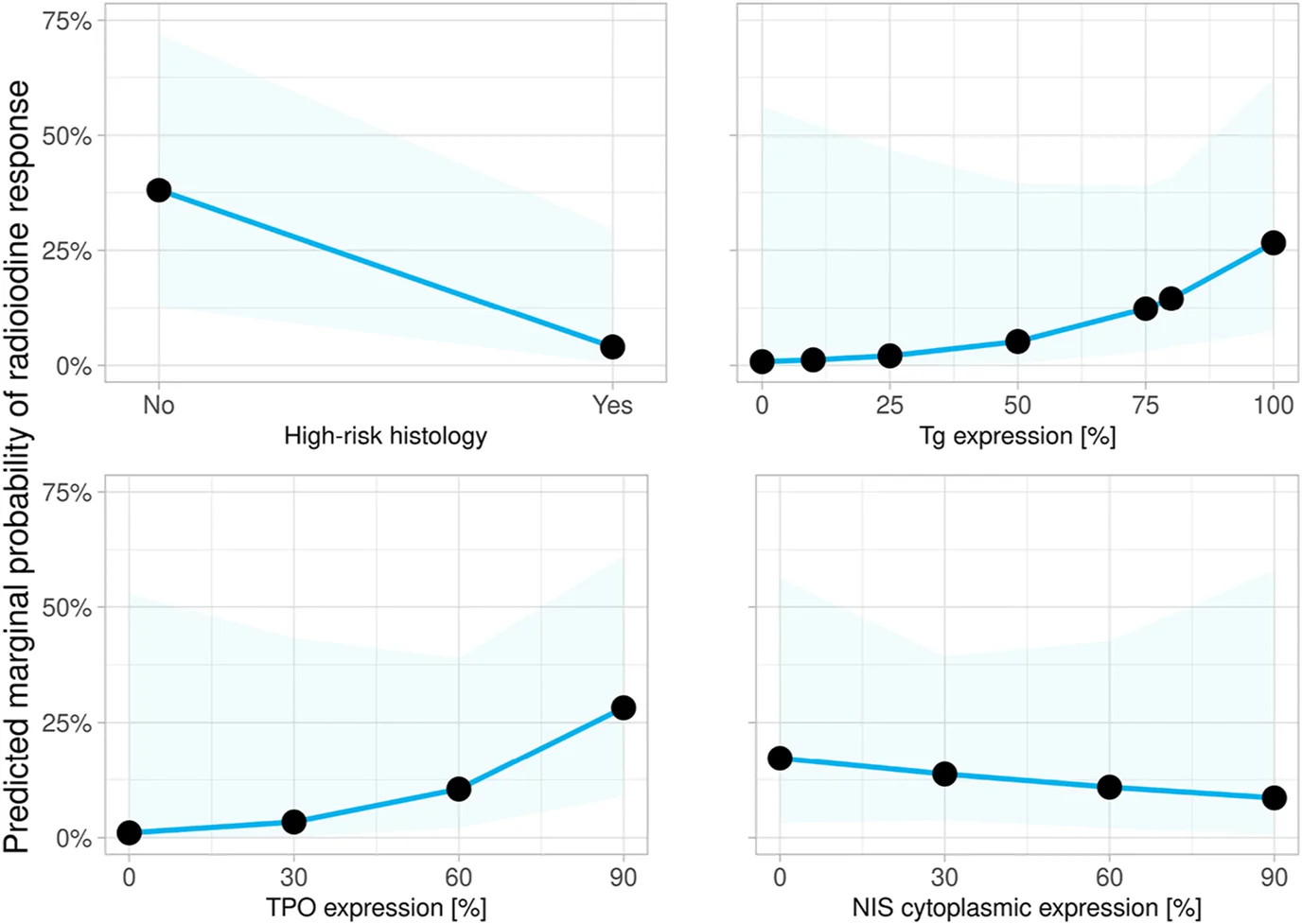
Marginal contributions to prediction of treatment response in the best-performing multivariate logistic regression model. Contributions of (top left) high-risk histology, (top right) Tg, (bottom left) TPO, and (bottom right) cytoplasmic NIS are shown. Confidence intervals (95%) are shown as a light blue shade. The numbers should be read as the probability of RAI treatment response depending on the plotted variable, given that the other three variables are set to the average value in the cohort. That is, if a patient has average TPO, Tg, and NIS expression, high-risk histology modifies the probability of response from 36% (if ‘No’) to 4% (if ‘Yes’). A full-color version of this figure is available at https://doi.org/10.1530/ERC-26-0253.

## Discussion

In this work, we aimed to identify which patients are most likely to receive sustained benefit from RAI therapy. While tumor stage and histological type excel at stratifying risk of recurrence and mortality, they alone showed limited ability to predict response to RAI in our cohort. Building on previous work in a prospective cohort of *ex vivo* quantified iodine concentrations in tumor tissue (27, 28), we have here validated predictors of response to RAI in patients with metastatic disease. The most consistent prediction was achieved by combining high-risk histology with immunohistochemical expression of TPO, Tg, and NIS. The results of this study may have several implications for RAI therapy.

First, the benefit of RAI administered as adjuvant therapy is under ongoing debate. A 2020 review showed mixed results and suggested only a modest adjuvant effect on disease progression and mortality (37). The 2025 ATA guidelines for thyroid cancer only recommend a consideration of RAI in patients with low-intermediate- and intermediate-high-risk disease (6). However, within those risk groups, subsets of patients will have highly iodine-avid microscopic tumors that eventually will recur. These are patients who likely benefit from adjuvant RAI. Our results suggest that immunohistochemical analysis and histological subtyping of surgical specimens can help identify such patients.

Second, in patients who present with distant metastases, the value of predicting iodine avidity from histopathological analysis is lower because pre- or post-therapeutic functional imaging already provides information to guide treatment decisions. However, there may still be added prognostic value in knowing the immunohistochemical profile of tumors most likely to have a long-term treatment effect.

A third implication relates to the use of selective inhibitors to enhance iodine avidity prior to RAI administration (redifferentiation), an approach currently being evaluated in multiple clinical trials (38). While patient selection for these therapies is currently primarily guided by specific molecular alterations (e.g. *BRAF* or *RET*) (17, 39), the results of the current study imply that genetic status alone does not fully predict iodine uptake capacity. For successful redifferentiation, functional reactivation of the iodide-handling machinery is required, and protein-level markers such as NIS or TPO may provide complementary information by reflecting the actual differentiation state and iodine-handling capacity of tumor cells. Accordingly, the markers for tumors unlikely to respond adequately to RAI therapy validated in this work may guide the selection of candidates for redifferentiation. Initiating such treatment before patients have progressed after serial RAI therapy may in turn improve the outcome of redifferentiation strategies.

In the current work, few cases had detectable membranous NIS expression, consistent with previous studies (18, 19, 20). Instead, cytoplasmic NIS expression was negatively correlated with iodine avidity and likelihood of treatment response. Mislocalization of NIS protein to the cytoplasm rather than the basolateral plasma membrane likely reflects a dysfunction in cellular signaling. One explanation may be that larger amounts of NIS present in the cytoplasm reflect an aberrant subcellular localization caused by unknown factors linked to poor treatment response. Interestingly, tumors with cytoplasmic NIS but without membranous NIS had among the lowest tumor-to-normal ratios (Supplementary Fig. S5). This finding would benefit from being revisited in further studies on treatment response and NIS localization in thyroid cancer tissue.

Our findings further suggest that assessing TPO and Tg expression, complemented by high-risk histology and possibly cytoplasmic NIS, is superior for predicting iodine avidity status compared with *BRAF* and *TERT* promoter mutations. Others have convincingly described such mutations as indicative of risk for RAI-refractory disease (40, 41, 42). This discrepancy may be due to an inherent superiority of TPO, Tg, and high-risk histology in predicting avidity. TPO expression (quantified as the proportion of cells with expression) may be an aggregate differentiation marker that more closely captures the iodine-handling machinery in tumor cells. Another explanation may be the fact that mutational status is a binary parameter, limiting the amount of information and therefore predictive power that can be conveyed by it as a predictor. In this work, immunohistochemical expression was scored as a percentage of cells, naturally allowing for more nuance in stratifying tumors. It is notable that high-risk histology was a more dominant factor in predicting RAI response, while TPO was the most prominent factor in predicting iodine avidity (Table 2), suggesting roles in different facets of thyroid cancer biology. The results may help bridge the gap between which markers to apply in clinical practice and knowledge of the underlying thyroid cancer biology, as iodine avidity becomes more thoroughly understood (43).

The limitations of this study include its retrospective design. Although most variables have been prospectively registered, this approach may introduce some bias. Iodine avidity was evaluated using tumor-to-normal ratios on post-therapy scans. This is used as a proxy measure for absorbed dose, which is more likely to be directly linked with treatment response. Importantly, the observed associations between iodine avidity and the identified predictors are consistent with findings from previous work (27, 28). The outcome variable of biochemical response was available in 20 of 31 patients, which may introduce bias in the evaluation. The reasons for not receiving a second RAI treatment were proportionally similar to the response rates overall (with approximately 30% responding well and the others lacking a sustained response to RAI). This suggests that limited, if any, bias was introduced by restricting the analysis to the 20 evaluable patients for that endpoint. Furthermore, the use of anti-Tg antibodies as a proxy in three out of the 20 evaluable patients may have introduced further potential bias.

Other limitations may arise from the inclusion of both well-differentiated and poorly differentiated tumors, as well as lymph node and distant metastases within the same cohort. However, sensitivity analyses performed to explore whether this could have biased the modeling found that the predictors and effect sizes remained similar despite excluding PDTCs and lymph node metastases. The biological differences between lymph node and distant metastases did not appear to strongly impact how they responded to RAI when TPO, Tg, NIS, and high-risk histology were taken into account. Furthermore, tumor biology in the primary and metastatic sites may be different due to intratumoral heterogeneity or successive dedifferentiation in either site, contributing to the unexplained variance in iodine avidity in the model. This could possibly limit the eventual value of using primary tumor samples to predict metastatic behavior, despite the results reported here. In addition, this study did not include broader genetic profiling, including fusion drivers, which may have been promising predictors of RAI response. However, the study would be underpowered to explore the effect of more uncommonly mutated genes. A larger external validation of the reported predictors, as well as more genetic markers, would further enhance the reliability of the results.

## Conclusion

Treatment response to RAI therapy and iodine avidity in thyroid cancer can be predicted by histological subtyping and immunohistochemical assessment of TPO, Tg, and NIS. Together, these markers explained more than half of the variation in iodine avidity and likelihood of RAI response. Furthermore, these markers outperformed conventional predictors such as pT stage, as well as mutational status of *BRAF* and the *TERT* promoter. Incorporating this information into routine pathological evaluation may therefore support more informed decision-making for both adjuvant and metastatic treatment strategies in thyroid cancer.

## Supplementary materials



## Declaration of interest

All authors declare that there is no conflict of interest.

## Funding

VC, MS, and DT received no related funding for this work. CL and CCJ received funding from the Swedish Cancer Society and the Stockholm County Council. JNN received funding from the Stockholm County Council.

## Author contribution statement

VC collected and analyzed the data and wrote the manuscript. MS collected the data. DT analyzed the data. CL analyzed data. CCJ designed the study, collected and analyzed the data, and wrote the manuscript. JNN designed the study, collected and analyzed the data, and wrote the manuscript. All authors approved the final version of the manuscript.

## Data availability

The data presented in this study are available upon reasonable request through the corresponding author.

## Institutional review

All participants provided written informed consent, and the study was approved by the Swedish Ethical Review Authority (EPM 2020-01541).

## AI disclosure

No generative artificial intelligence (AI) tools were used in the preparation of the written content of this manuscript. Some AI assistance was used in coding for illustrations in this manuscript.
