# Supplementary material for: Predicting radioiodine therapy response in advanced and recurrent thyroid cancer
Source: Endocr Relat Cancer. 2026 Sep 29;33(9):e260253. doi: 10.1530/ERC-26-0253 (PMC13641125; doi:10.1530/ERC-26-0253)
Supplement: Supplementary file 1 [file ERC-26-0253_supplementary_materials.pdf]

Supplementary material

Figure S1. Absorbed dose vs tumor-to-normal signal ratio (n=6). For the six patients in which post-therapeutic dosimetry was performed, there was excellent agreement between the two variables aiming to reflect iodine avidity in tumor tissue. Patients undergoing dosimetry were administered 7.4 GBq ^{131}I.

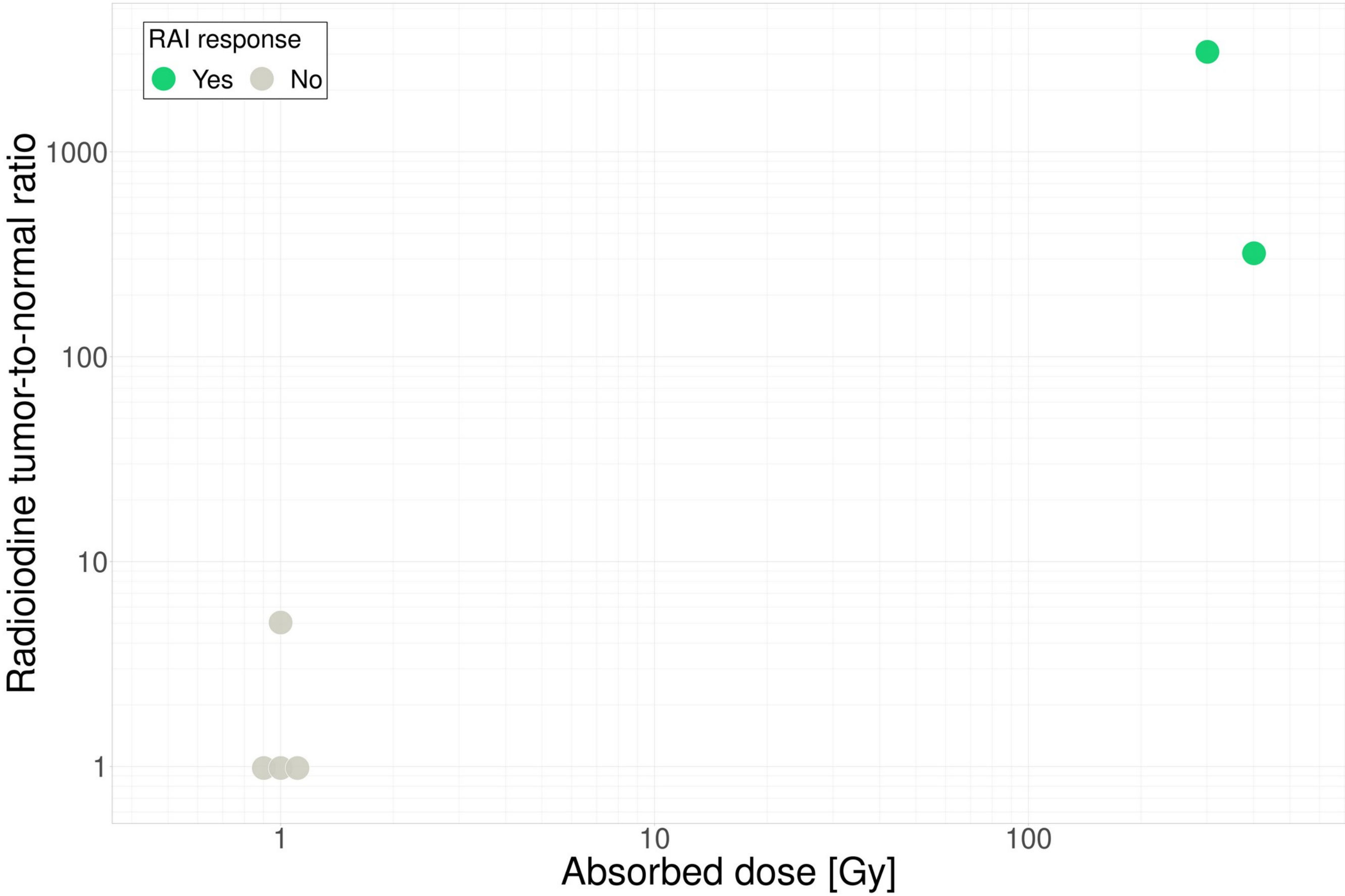

Figure S2. Tumor-to-normal signal ratio vs serum Tg change (n=20). A significant negative correlation was observed between the two variables, indicating agreement between iodine avidity and biochemical response to treatment.

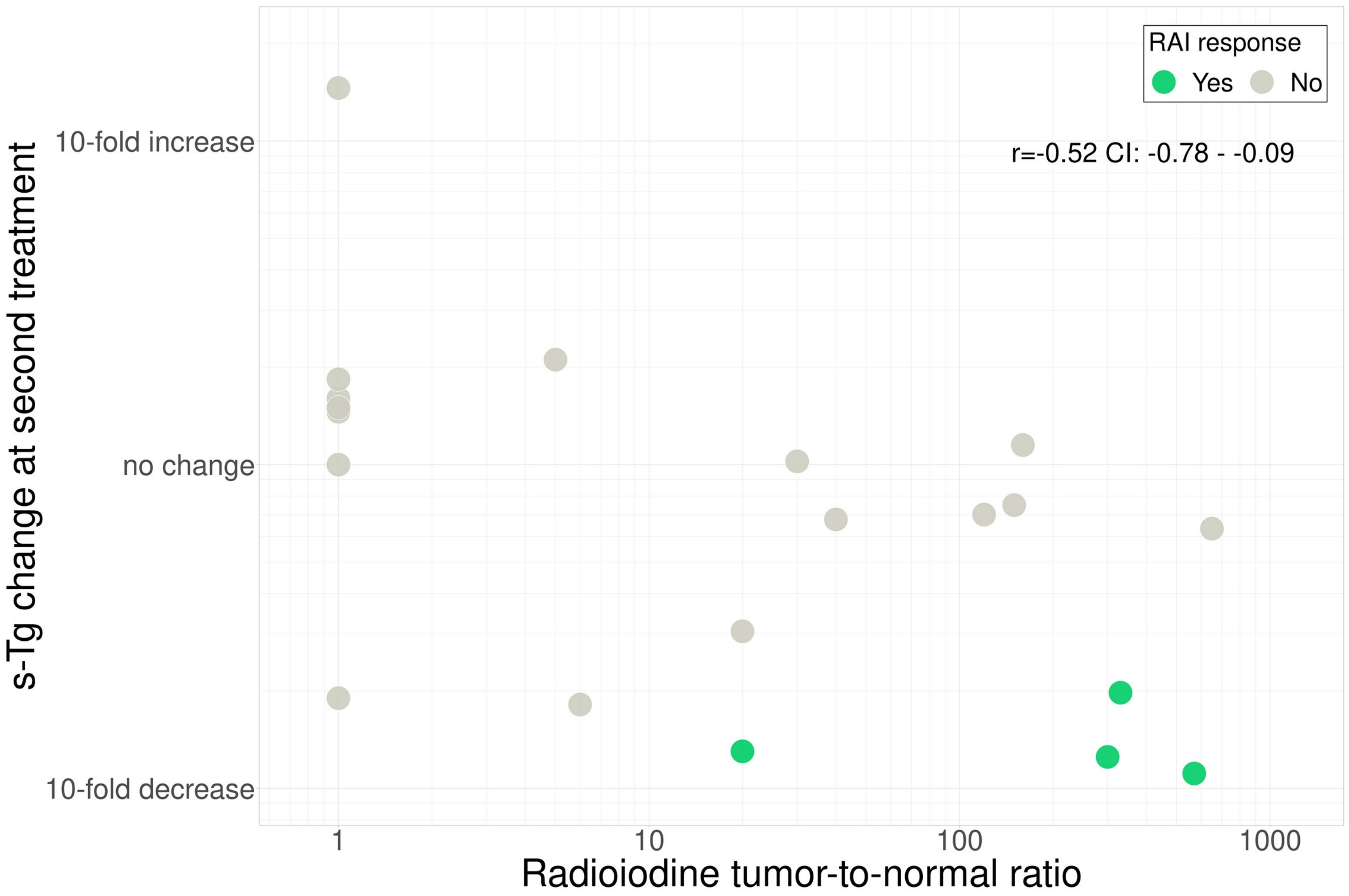

Figure S3. Thyroid cancer histological type vs tumor-to-normal ratios and RAI response (n=31). No significant difference in tumor-to-normal ratios or response between FTC and PTC was observed. Due to a low number of cases, further statistical analysis was not performed on FT-UMP, DHGTC or PDTC. FTC = follicular thyroid carcinoma; PTC = papillary thyroid carcinoma; FT-UMP = follicular tumor of unknown malignant potential; DHGTC = differentiated high-grade thyroid carcinoma; PDTC = poorly differentiated thyroid carcinoma.

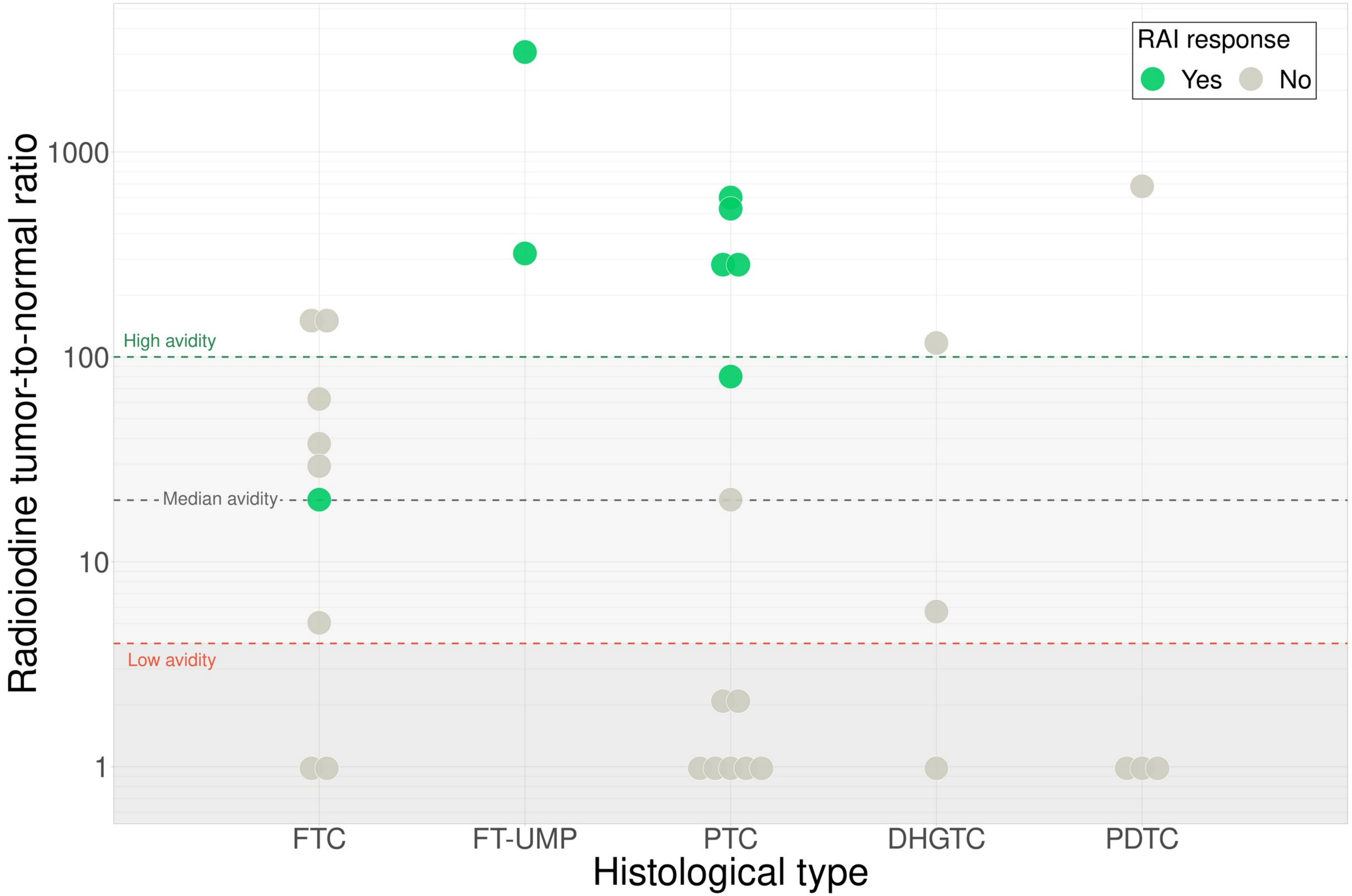

Figure S4. Ki67 index vs tumor-to-normal ratios and RAI response (n=31). No significant correlation was found between Ki67 index and tumor-to-normal ratio, and no significant difference in Ki67 was observed when comparing those with overall response and those without.

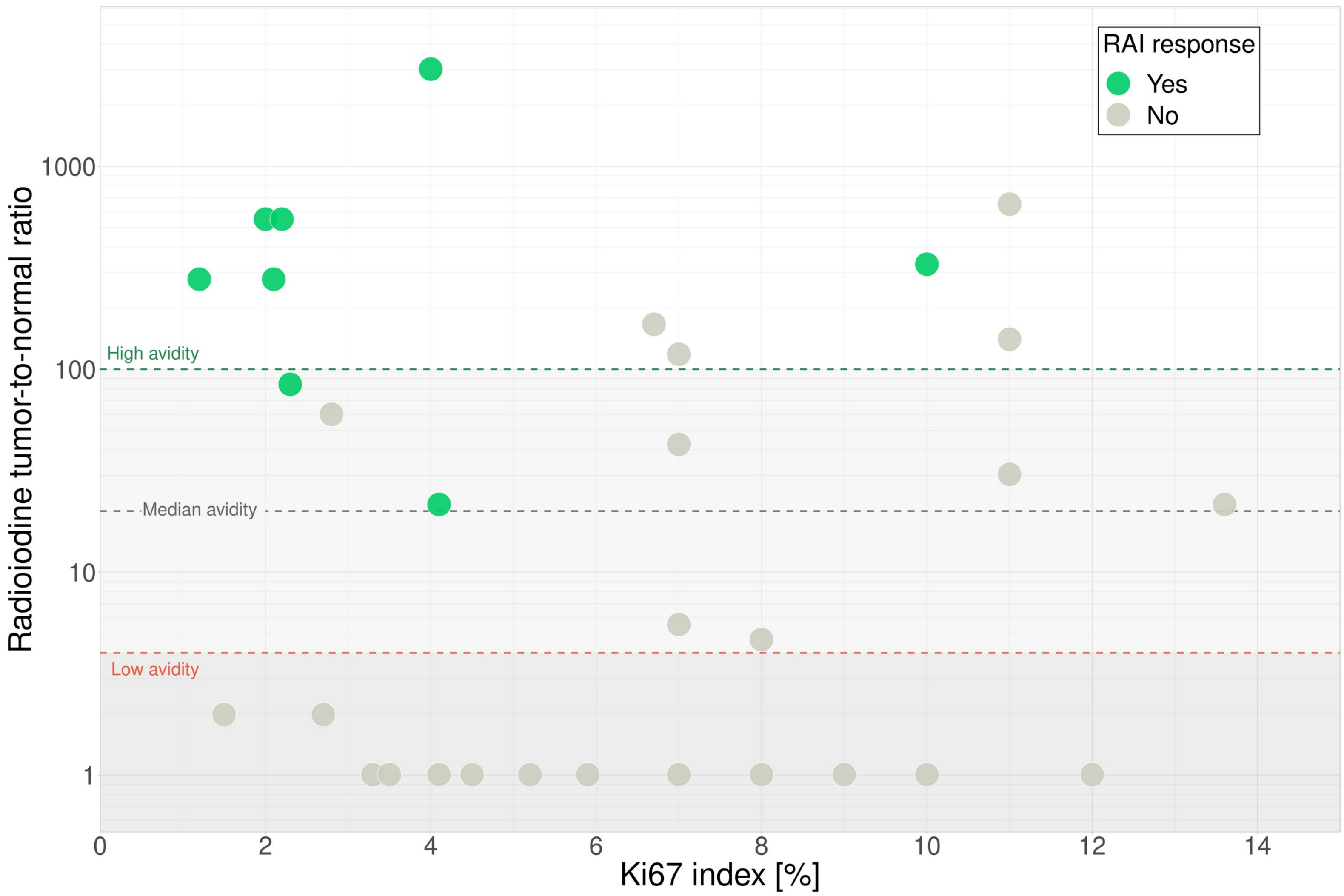

Figure S5. Tumor-to-normal ratios vs NIS cytoplasmic and membranous expression (n=31). Most cases with poor tumor-to-normal ratios had no membranous expression of NIS. However, the highest tumor-to-normal ratios were found in tumors with both cytoplasmic and membranous NIS expression.

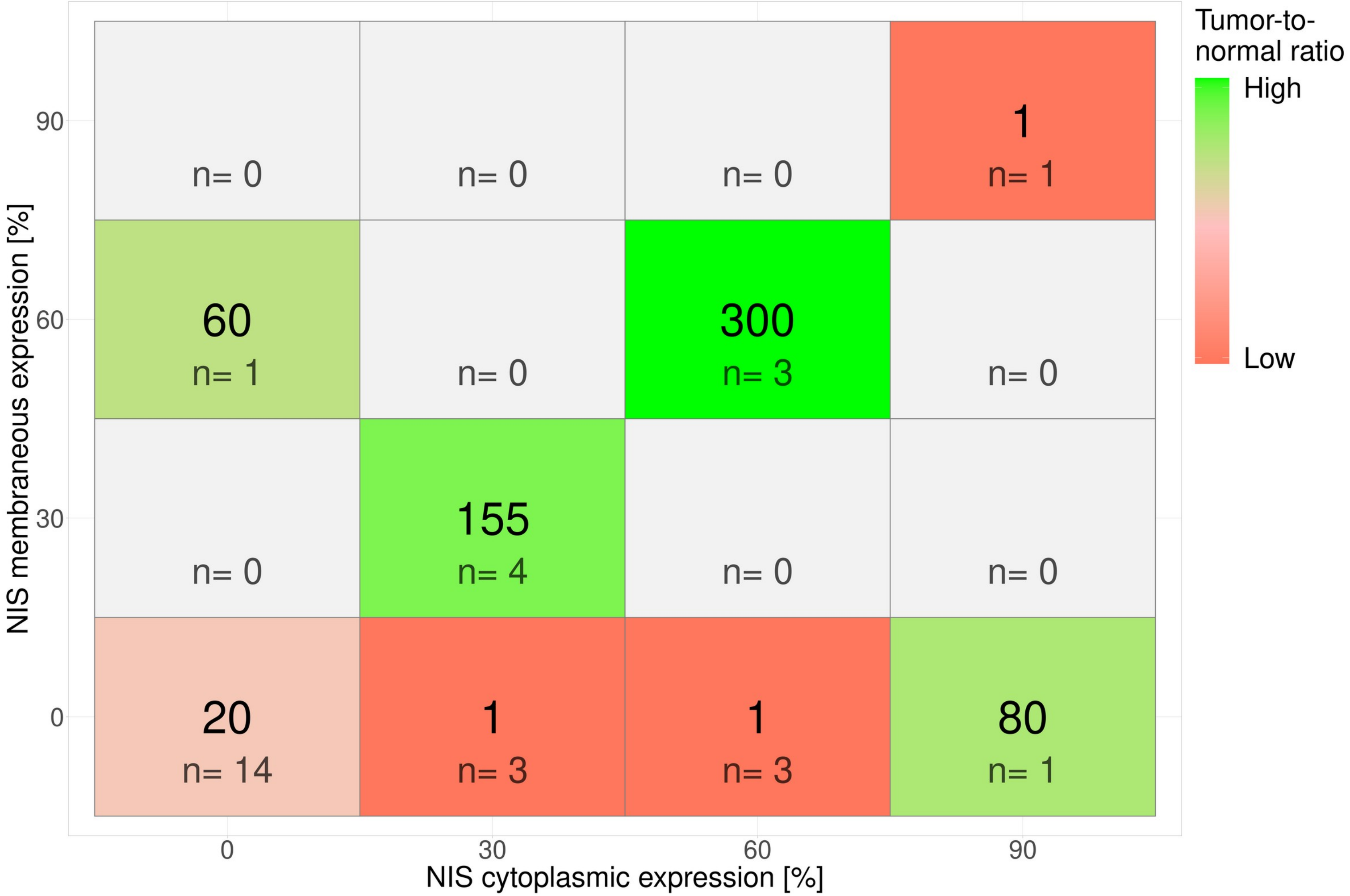

Supplementary Table S1. Multivariate modelling results with “stable disease” classified as RAI-responders.

Predicted endpoint	Predictive variable	exp(β)	95% CI	
**RAI response (logistic regression)**		(odds ratio)		Pseudo-R2: 0.19
Sustained biochemical and structural response	Intercept: odds 0.12			
	High-risk histology [yes]	0.16	0.02-0.91*	
	TPO expression [per 10 %]	1.32	0.97-1.99	
	Cytoplasmic NIS expression [per 10 %]	0.80	0.54-1.11	
	Tg expression [per 10 %]	1.13	0.82-1.68	

The numbers in the exp(β) columns are the estimated odds modifiers.
P-values: * <0.05
